# The use of polyhydroxylated carboxylic acids and lactones to diminish biofilm formation of the pathogenic yeast *Candida albicans*†

**DOI:** 10.1039/c9ra01204d

**Published:** 2019-04-09

**Authors:** Olena P. Ishchuk, Olov Sterner, Helena Strevens, Ulf Ellervik, Sophie Manner

**Affiliations:** Department of Biology, Lund University Sölvegatan 35 SE-223 62 Lund Sweden; Centre for Analysis and Synthesis, Centre for Chemistry and Chemical Engineering, Lund University P. O. Box 124 SE-221 00 Lund Sweden sophie.manner@chem.lu.se; Department of Obstetrics and Gynecology, Skånes Universitetssjukhus SE-221 85 Lund Sweden

## Abstract

The vaginal microbiome of healthy women is a diverse and dynamic system of various microorganisms. Any sudden change in microbe composition can increase the vaginal pH and thus lead to vaginal infections, conditions that affect a large percentage of women each year. The most common fungal strains involved in infections belong to the yeast species *Candida albicans*. The main virulence factor of *C. albicans* is the ability to transform from planktonic yeast-form cells into a filamentous form (hyphae or pseudohyphae), with the subsequent formation of biofilm. The hyphal form, constituted by filamentous cells, has the ability to invade tissue and induce inflammation. Our hypothesis is that certain polyhydroxylated carboxylic acids, that may serve as an alternative carbohydrate source and at the same time lower the pH, function as an indicator of a nutrient-rich environment for *C. albicans*, which favors planktonic cells over hyphae, and thus diminish the formation of biofilm. We have shown that the biofilm formation in *C. albicans* and other *Candida* species can be significantly reduced by the addition of glucono-δ-lactone (GDL).

## Abstract 2

Treatment of *Candida albicans* with glucono δ-lactone results in less formation of hyphae and diminish biofilm formation.

## Graphical abstract

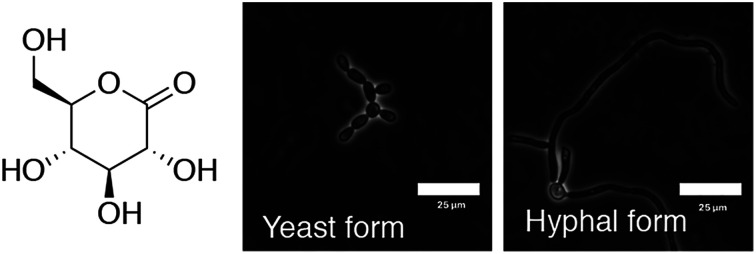

## Introduction

The vaginal microbiome is a diverse and dynamic system of various microorganisms. This microbial community depends largely upon *Lactobacillus* species that produce lactic acid and maintain the weakly acidic environment (typically pH 3.5 to 4.5) of healthy individuals. The exact composition of *Lactobacillus* species may be important to explain the propensity for some women to contract health problems such as viral, bacterial, or fungal pathogen infections, as well as premature deliveries.^1,2^ A sudden change in the vaginal microflora can increase the vaginal pH and consequently create a more favorable environment for the establishment of vaginal pathogens, which grow optimally at a pH over 5. The vaginal infections caused by these pathogens affect a large percentage of women of reproductive age each year. The most common strains, causing 85–95% of all fungal infections, belong to the species *Candida albicans*.^3,4^

Vaginal *Candida* infection is a common problem, affecting most women at times. Between 70–75% of all women are believed to be affected at least once in their lifetime, while approximately 5–8% experience extremely bothersome and recurrent infections.^3^ The symptoms include itching, soreness or irritation, reddened and swollen vaginal tissues, pain with urination and intercourse, typically adhesive white and clumpy discharge or normal to thin and watery discharge. *C. albicans* is normally present in smaller amounts in the vagina, mouth, digestive tract, and on the skin of healthy individuals without causing infection, but with changes in the normal microflora, caused for example by antibiotic treatments, *C. albicans* can become more abundant and cause infections. Data concerning the precise occurrence of vulvovaginal infections is likely to be incomplete due to the psychosocial stigma associated with genital infections, and the numbers may well be higher than previously described.^3,4^

*C. albicans* thrives on the glycogen present in vaginal mucosa, and infections are facilitated by the effect of increased estrogen levels on the mucosa during pregnancy as well as by the weakened immune system during gestation. Contraceptive pills, menstruation, diabetes, and other stress factors can also enhance the occurrence of the infections.^3^

Although vulvovaginal candidiasis is not a life-threatening condition, it can become chronic and thus reduce the quality of life, sex life, work, and the ability to concentrate on immediate tasks; a chronic condition can eventually lead to depressions. The chronic condition can also cause debilitating vestibulitis, which can be exceedingly difficult to treat.^5^

It is estimated that approximately 80% of infections in humans, including vulvovaginal *Candida* infections, are related to the formation of biofilm, *i.e.* the formation of complex three-dimensional structures of the pathogens bound to host cell walls as well as to other pathogen cells.^6^ The formation of biofilm also reduces the efficiency of anti-fungal drugs by 10–100 times. It has been shown that biofilm formation is required for vulvovaginal *Candida* infections.^6^ The prerequisite for biofilm formation by *C. albicans* is the morphological transition of planktonic yeast-form cells into filamentous hyphae or pseudohyphae, which have increased adhesion properties.^7^ The hyphal form also has the ability to invade tissue and induce inflammation, which is mediated by candidalysin, a cytotoxic peptide toxin that facilitates the penetration of the hyphae into the epithelial cells.^8^

Current treatment of *Candida* infections include topical application or oral administration of azole antifungals, such as fluconazole.^3^ However, although side effects of fluconazole are mild and infrequent (stomach upset, headache and rash), fluconazole may interact with a number of medications and is not recommended during pregnancy due to the potential risk of harm to the fetus. Alternative treatments of vulvovaginal candidosis involve the use of lactic acid and lactic acid bacteria.

Our hypothesis is that certain polyhydroxylated carboxylic acids, that may serve as alternative carbohydrate sources and at the same time lower the pH, may function as a trigger of a favorable environment for *C. albicans*, which favors planktonic yeast-form cells over the hyphal form, and thus diminish the formation of biofilm.

## Experimental

dl-Lactic acid, dl-glyceric acid, d-xylonic acid, d-gluconic acid, citric acid, and d-glucono-δ-lactone were obtained from commercial suppliers. The following *Candida* strains were used: *C. albicans* SC5314,^9^*C. glabrata* CBS138,^10^*C. tropicalis* silicone isolate U3-3 (Atos Medical AB), *C. tropicalis* silicone isolate A6-1 (Atos Medical AB), *C. krusei* silicone isolate U3-5 (Atos Medical AB), *C. krusei* silicone isolate U2-12 (Atos Medical AB), *C. krusei* silicone isolate A5-2 (Atos Medical AB), and *C. krusei* silicone isolate A4-1 (Atos Medical AB).

### Biofilm formation assay

Yeast strains were grown at 37 °C in complete medium YPD (0.5% (weight/volume) yeast extract, 1% (weight/volume) peptone, 2% (weight/volume) glucose) or minimal medium consisting of YNB (yeast nitrogen base without amino acids and ammonium sulphate, FORMEDIUM™, CYN0505) supplemented with 0.45% (weight/volume) ammonium sulphate, 0.2% (weight/volume) glucose and 100 mM l-proline. If needed, 2% (weight/volume) agar was used to solidify media. The liquid minimal medium (YNB (yeast nitrogen base without amino acids and ammonium sulphate, FORMEDIUM™, CYN0505) supplemented with 0.45% ammonium sulphate, 0.2% (weight/volume) glucose and 100 mM l-proline) was used for biofilm assay (biofilm medium). In the experiments on the impact of different acids, lactic acid, glyceric acid, xylonic acid, gluconic acid, or citric acid were added to a final concentration of 0.06% (weight/volume). In the experiments on the impact of pH on biofilm the pH values (from 2.6 to 6.6) were obtained using either different potassium phosphate buffers at the final concentration 0.25 M, or by the addition of lactic acid, citric acid, gluconic acid, or GDL to the biofilm medium.

Biofilm was measured in liquid culture as described^11,12^ with some modifications. Prior the biofilm assay, yeast cultures were grown in liquid YPD medium for 24 h until stationary phase. Cells were then pelleted by centrifugation (1699 × *g*), washed with sterile MQ water and the cells were further inoculated into test biofilm medium (YNB (yeast nitrogen base without amino acids and ammonium sulphate) supplemented with 0.45% ammonium sulphate, 0.2% glucose and 100 mM l-proline pH 7.0) at final concentration of 0.2 OD_600_ mL^−1^ and incubated in 96-well flat-bottom polystyrene microtiter plates (Sigma Aldrich, Corning® Costar® culture plates, CLS3596-50EA) for 72 h at 37 °C. At defined time points crystal violet (HT901-8FOZ; Sigma Aldrich) was added to the media at the final concentration 0.05%. After 24 h of cells staining, plate wells were washed four times with 200 μL of water to remove planktonic (non-adherent) cells. Biofilms were then dried and dissolved in 200 μL of 96% ethanol. In addition, total biomass (biofilm and planktonic cells) was measured spectrophotometrically in unstained wells; for this the cells in the well were re-suspended by pipetting (to obtain both biofilm and planktonic cells). Optical density (OD) measurements of both total biomass and crystal violet biofilm after staining were performed at 560 nm with FLUOstar OPTIMA plate reader, BMG LABTECH spectrophotometer. Crystal violet biofilm measurements were normalized to the total biomass (OD_560_biofilm/OD_560_total biomass). For investigations of mature biofilm, the biofilm was allowed to grow for 48 h. Then, medium and planktonic cells were removed and new media added.

### Biofilm viability assay

Viability of biofilms after treatment with GDL at different concentrations and different time periods was evaluated by an improved XTT method.^13^ The mature biofilm was exposed to GDL for 24 h. Then the cells were washed 2 times with PBS to remove planktonic cells, after which 200 μL of reaction mixture was added to each well of microtiter plate to adherent cells: PBS buffer containing 200 mM glucose, 0.2 mM XTT (2,3-bis(2-methoxy-4-nitro-5-sulfophenyl)-5-[(phenylamino)carbonyl]-2*H*-tetrazolium hydroxide, X4626, Sigma-Aldrich), and 4 μM menadione.^13^ After 0.5 h of incubation in the dark, the optical density was measured at 485 nm. The viable cells reduce the XTT to colored formazan.

### Sensitivity to calcofluor white as indicator of cell wall damage

To deduce cell wall damage,^14^ cells from biofilm experiments were plated onto YPD solid media with calcofluor white at 10 and 70 μg mL^−1^ with or without addition of 0.5 M sucrose (osmotic stabilizer) and incubated at 37 °C.

### Microfluidics study of biofilm development

Microfluidics plates (CellASIC® ONIX Y04D-02-5PK, Merck Millipore) were used with ONIX Microfluidic Perfusion System and were inoculated with yeast at 8 psi for 5 s according to manufacturer recommendations, flowed at 1.5 psi with media tested. Hyphae started to form within first hour of incubation in the biofilm medium (YNB supplemented with 100 mM l-proline and 0.2% glucose, 0.45% of ammonium sulphate, pH 7.0). GDL (2.5 g) was added to buffer solution of pH 3.71 (0.5 M KH_2_PO_4_/*ortho* phosphoric acid, 10 mL) at 37 °C. A sample was taken after 1 h and diluted 50 times with biofilm medium and added to *C. albicans*. The yeast growth and biofilm development were monitored over time on fully motorized and automated inverted widefield microscope Observer Z1 (Carl Zeiss) equipped with a sCMOS camera. The phase-contrast images were taken over time specified.

### Growth on YPD to estimate cell viability

Biofilm of *C. albicans*, was grown for 48 h in YNB, 0.2% glucose, 0.45% ammonium sulphate, 100 mM l-proline, pH 7.0. Then the biofilm medium was removed and GDL of different concentrations (0.05–0.5 g mL^−1^) added at 37 °C. After incubation with GDL for 5 h, 5 μL of cells were plated at serial dilution (1 : 10 to 1 : 1000) on the agar medium YPD. The plated cells were incubated for 24 h at 37 °C and visually analyzed.

### Hydrolysis of GDL

In water solution GDL is in equilibrium with gluconic acid. GDL (200 mg) was added to 20 mL of pH 4, 5 or 7 buffers (0.1 M citric acid/0.2 M Na_2_HPO_4_) at 37 °C. Optical rotation, measured at 37 °C, sodium D line, *C* = 10 mg mL^−1^, path length = 10 cm.

### Statistical analysis

The software package Minitab® 18.1, were used to analyse the obtained data.

## Results

### Organic acids lower pH and reduce the biofilm formation

**Chart 1:**
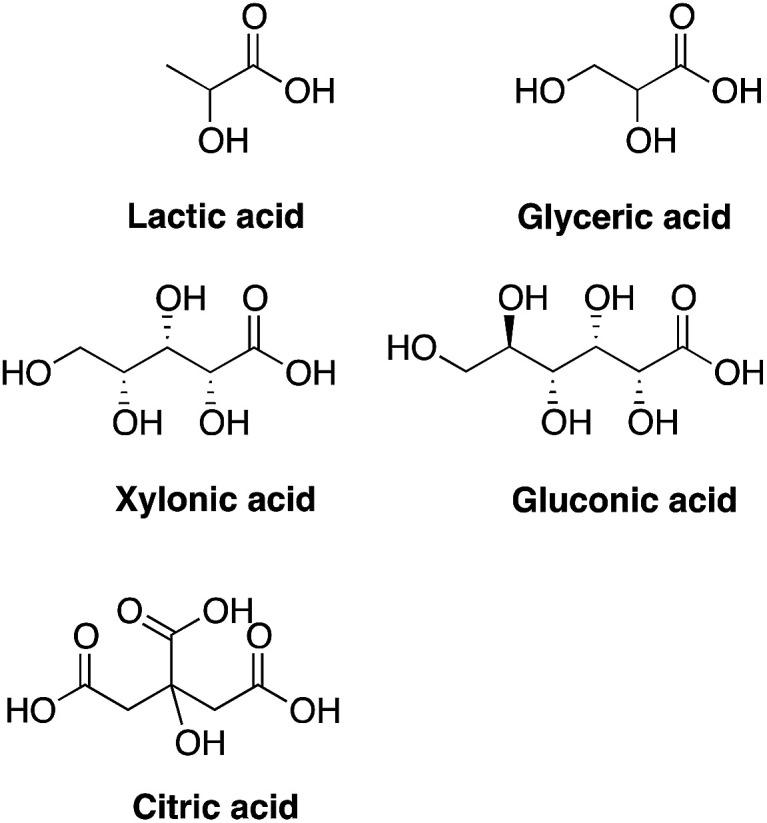
Structures of tested compounds.

**Fig. 1:**
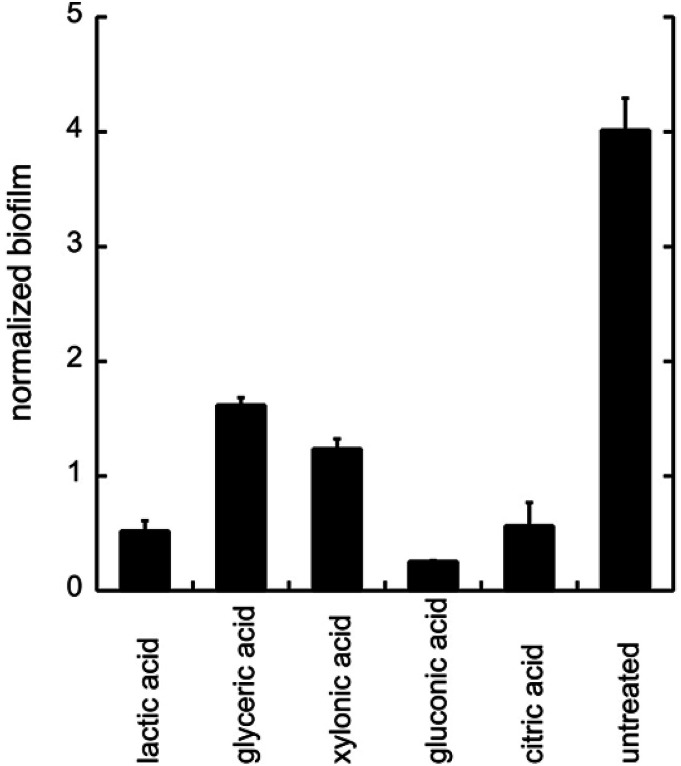
The biofilm formation of *C. albicans* with the addition of different organic acids. Biofilm was treated with different acids (0.06 wt%) under unbuffered conditions. The biofilm was measured after 24 h of addition of acids by crystal violet staining. All experiments were performed in triplicate.

To evaluate the effects of low concentrations of different polyhydroxylated carboxylic acids, the formation of biofilm was measured in liquid culture,^11,12^ using *Candida albicans* SC5314 (ref. 9) and the addition of 0.06 wt% of lactic acid, glyceric acid, xylonic acid, gluconic acid, and citric acid (Chart 1), under unbuffered conditions. The formation of biofilm was measured after 24 h, using the crystal violet method^11^ and normalized to the total biomass of both planktonic and biofilm cells (OD_560_biofilm/OD_560_biomass). The data are shown in Fig. 1.

As biofilm formation is dependent on both pH and the occurrence of alternative energy sources, it was not surprising that lactic acid affected the formation of biofilm of *C. albicans* (Fig. 1). In addition, other polyhydroxylated C3–C5 carboxylic acids diminished the formation of biofilm. Surprisingly, we found that gluconic acid, *i.e.* a polyhydroxylated C6 carboxylic acid, provided superior effects in decreasing the formation of biofilm, in spite of being less acidic compared to, for example, citric acid (Chart 1; Fig. 1).

**Fig. 2:**
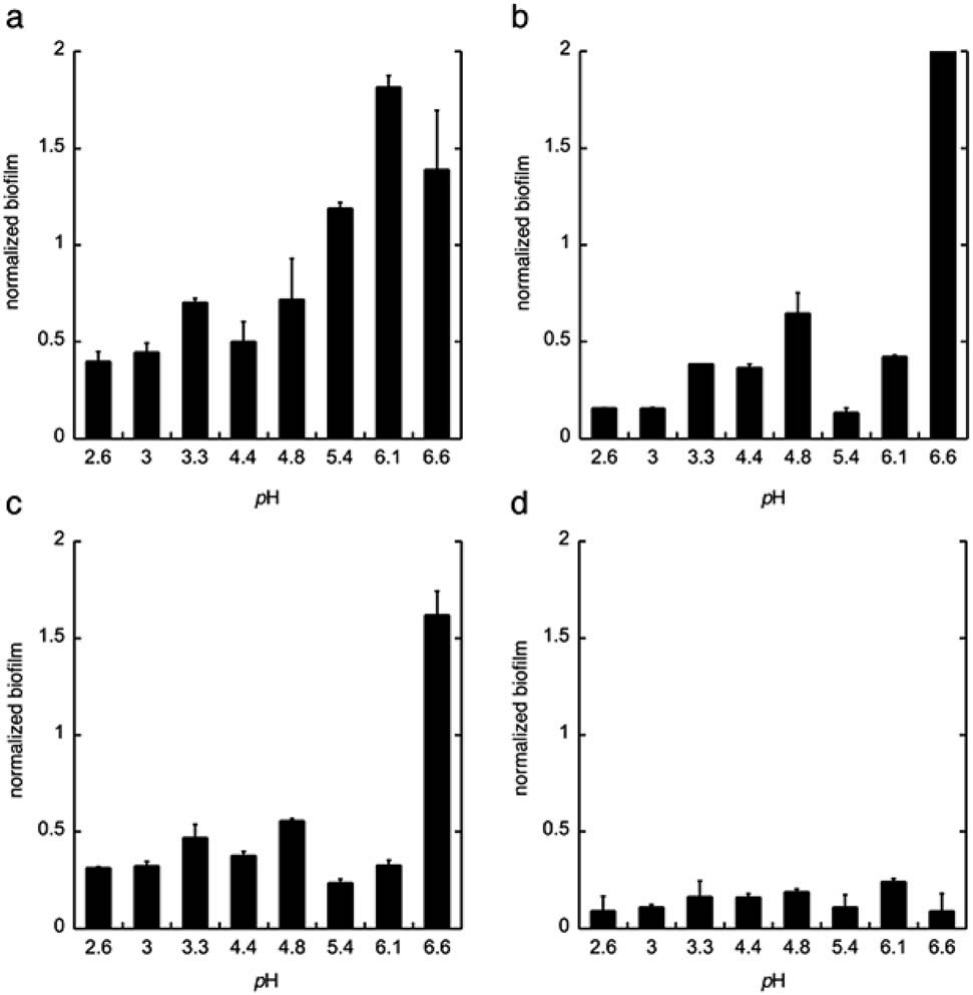
The biofilm formation of *C. albicans* in the minimal media at pH 2.6–6.6 with (a) phosphate buffer, (b) citric acid, (c) lactic acid, and (d) gluconic acid. The biofilm was measured after 24 h. Biofilm staining was performed with crystal violet. All experiments were performed in triplicate.

Next, citric acid, lactic acid, and gluconic acid were added under unbuffered conditions to give a pH between 2.6 and 6.6, or buffered conditions (potassium phosphate buffer). The formation of biofilm was measured after 24 h, using the crystal violet method^11^ and normalized to the total biomass of both planktonic and biofilm cells (OD_560_biofilm/OD_560_biomass). The data are shown in Fig. 2.

From these data we conclude that gluconic acid, in contrary to citric acid and lactic acid, show a pronounced effect on the formation of biofilm by *C. albicans*, already at near neutral pH (6.6), which indicates that the effect of gluconic acid is not only due to a pH-lowering effect.

### Glucono-δ-lactone is suitable for pharmacological applications

**Scheme 1:**
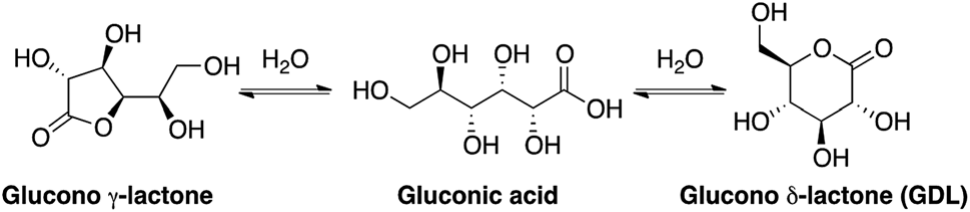
Structures of gluconic acid and the corresponding lactones.

Gluconic acid is difficult to produce as a solid crystalline product, and is usually supplied as a 50% water solution, which is less suitable for pharmaceutical compositions. However, in water solution gluconic acid is in equilibrium with the corresponding glucono-δ-lactone (GDL) and the glucono-γ-lactone (Scheme 1). GDL is a solid compound, which decomposes at 153 °C without melting.

**Fig. 3:**
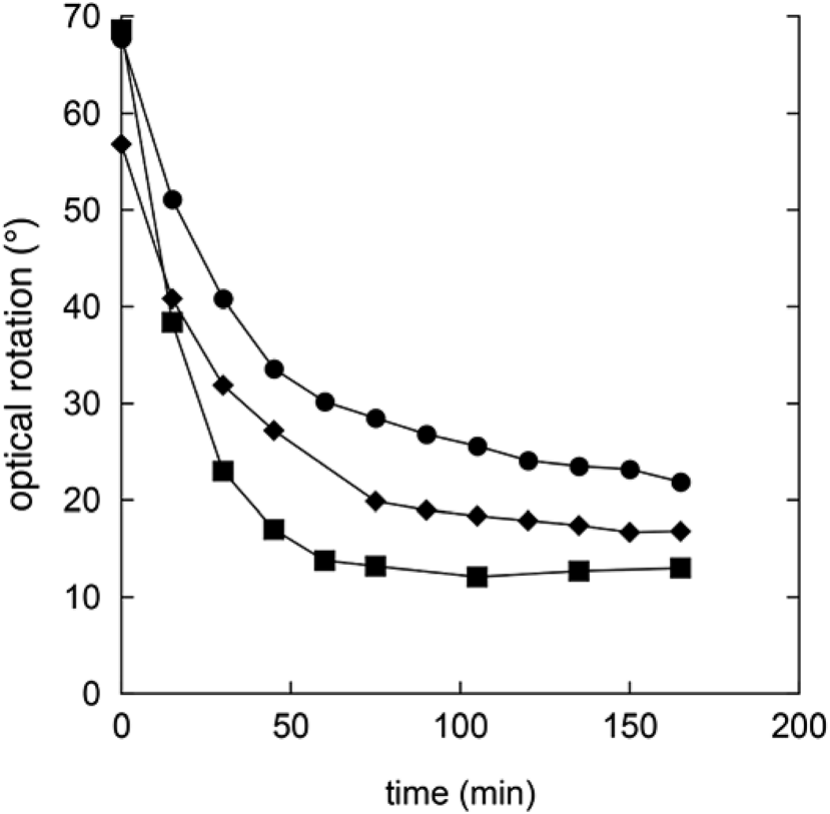
Changes in optical rotation in the hydrolysis of GDL pH 4 buffer (filled squares), pH 5 buffer (filled circles), and pH 7 buffer (filled diamonds).

In order to understand the rates and equilibrium concentrations of the GDL–gluconic acid system, GDL was added to a pH 4 buffer, a pH 5 buffer, or a pH 7 buffer at 37 °C and optical rotations were measured over time. The optical rotations of GDL and the corresponding γ-lactone, are approximately 66°, while the optical rotation of gluconic acid is approximately 5°.^15^ These data show that GDL is slowly hydrolyzed to a mixture of GDL and gluconic acid (Fig. 3). The equilibrium is pH-dependent and relevant concentrations of GDL are present at all buffered conditions. The data are in accordance with earlier investigations.^15–17^ To verify the exact composition of the equilibrium, a sample of gluconic acid (50% water solution) was dissolved in DMSO-*d*^6^ and analyzed by ^1^H- and ^13^C-NMR and showed 70% gluconic acid, 15% GDL, and 15% of the corresponding γ-lactone.

**Fig. 4:**
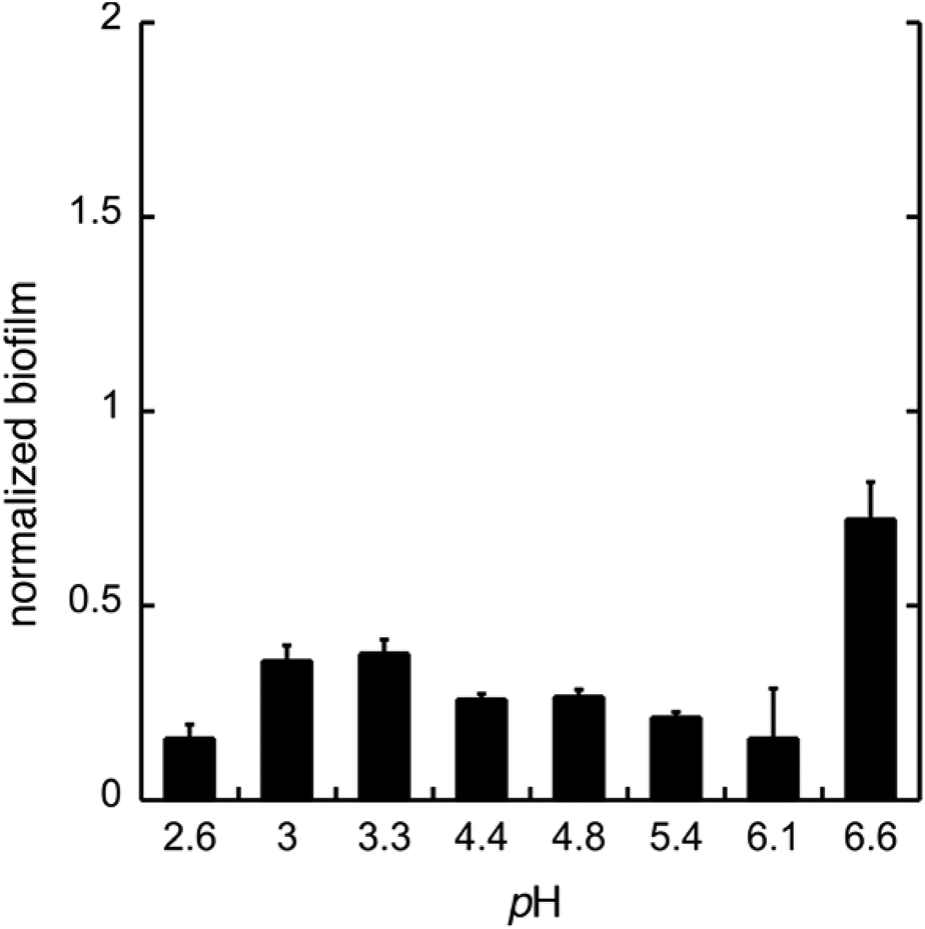
The biofilm formation of *C. albicans* in the minimal media at pH 2.6–6.6 with GDL. The biofilm was measured after 24 h. Biofilm staining was performed with crystal violet. All experiments were performed in triplicate.

To test whether GDL has similar effect on biofilm as gluconic acid, we used GDL in unbuffered conditions to give pH between 2.6 and 6.6. The formation of biofilm was measured after 24 h, using the crystal violet method.^11^ The data are shown in Fig. 4.

As expected, the effect of GDL is comparable to that of gluconic acid and a diminished formation of biofilm can be seen already at near neutral pH.

### GDL affects the morphological transition to hyphae form

**Fig. 5:**
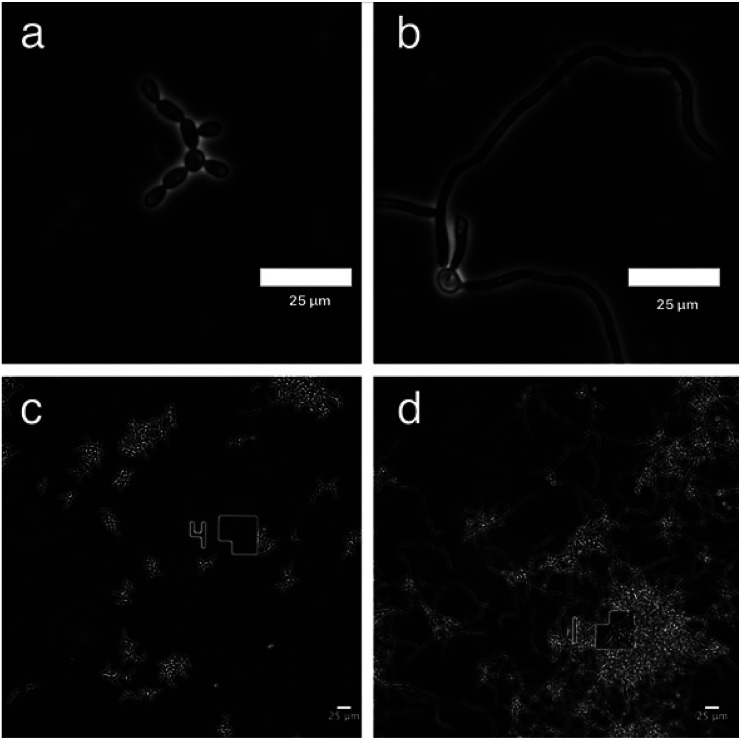
Microscope images of *C. albicans* show yeast-like morphology upon GDL treatment (a) and (c) *C. albicans* treated with GDL are mainly in yeast-form cells. (b) and (d) Untreated *C. albicans* cells are mainly in hyphal form.

To monitor the cell morphology of *C. albicans*, yeast cells were inoculated in the biofilm medium (YNB supplemented with 100 mM l-proline and 0.2% glucose, pH 7.0) with or without the addition of GDL (5 mg mL^−1^). In the untreated sample, hyphae started to form within the first hour of incubation, while treatment with GDL resulted in mainly yeast-form cells (Fig. 5). The process was also followed by time-lapse microscopy (ESI†) showing that the addition of GDL prevents the formation of hyphae.

### High concentrations of GDL inhibits *C. albicans* viability

**Fig. 6:**
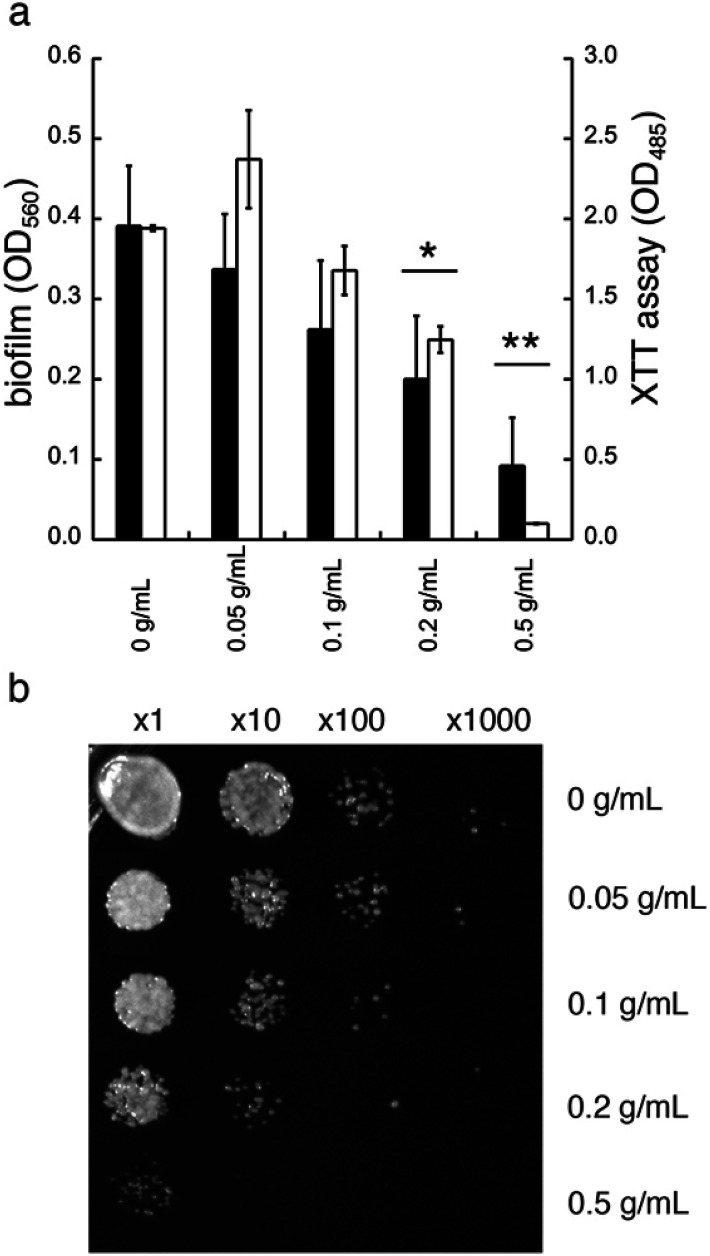
Treatment of mature (48 h) biofilm of *C. albicans* with GDL. (a) Filled columns indicate mature biofilm of *C. albicans* after treatment with GDL at different concentrations after 24 h of treatment. The biofilm staining was performed with crystal violet. Optical density was measured at 560 nm. Unfilled columns indicate the viability of biofilms of *C. albicans* after treatment with GDL at different concentrations for 24 h. The biofilm staining was performed with XTT. Optical density was measured at 485 nm. All experiments were performed in triplicate. (ANOVA *p-value ≤ 0.008 and 0.03 for 0.2 g mL^−1^ of GDL, and **p-value ≤ 0.0003 and 0.002 for 0.5 g mL^−1^ of GDL.) (b) The effect of GDL on mature biofilm of *C. albicans*. Mature biofilm was incubated with GDL for 5 h at 37 °C and then cells at serial dilution were plated on YPD plate to estimate cell survival.

In addition to diminished biofilm formation, GDL may also affect the cell viability of *C. albicans*. To study this, we first tested the effect of GDL on mature biofilm. The mature biofilm (grown for 48 h in YNB, 0.2% glucose, 100 mM l-proline) of *C. albicans* was incubated with GDL of different concentrations (0.05–0.5 g mL^−1^) at 37 °C and the formation of biofilm was measured after 24 h, using the crystal violet method. The data are shown in Fig. 6a. The mature biofilm was shown to be less sensitive to GDL, compared to the direct addition of GDL before biofilm formation. However, at high concentrations of GDL (*i.e.* 0.2 and 0.5 g mL^−1^), the biofilm formation was significantly decreased, which may indicate that at these concentrations, GDL may show a cytotoxic effect on *C. albicans*. To test this, the viability of biofilms of *C. albicans* after treatment with GDL at different concentration and different time periods was evaluated by staining the cells with XTT (a colorimetric assay for quantification of cellular viability).^13^

The XTT assay showed a decrease in viability for *C. albicans* at high concentrations of GDL (0.2 and 0.5 g mL^−1^, similar to crystal violet, Fig. 6a). Furthermore, the cytotoxic effect was observed already after 5 h of treatment. As shown in Fig. 6b, the GDL at 0.2 and 0.5 g mL^−1^ substantially reduced the colony forming units to approximately 10-fold (for 0.2 g mL^−1^) and 100-fold (for 0.5 g mL^−1^) compared to untreated control (Fig. 6b).

**Fig. 7:**
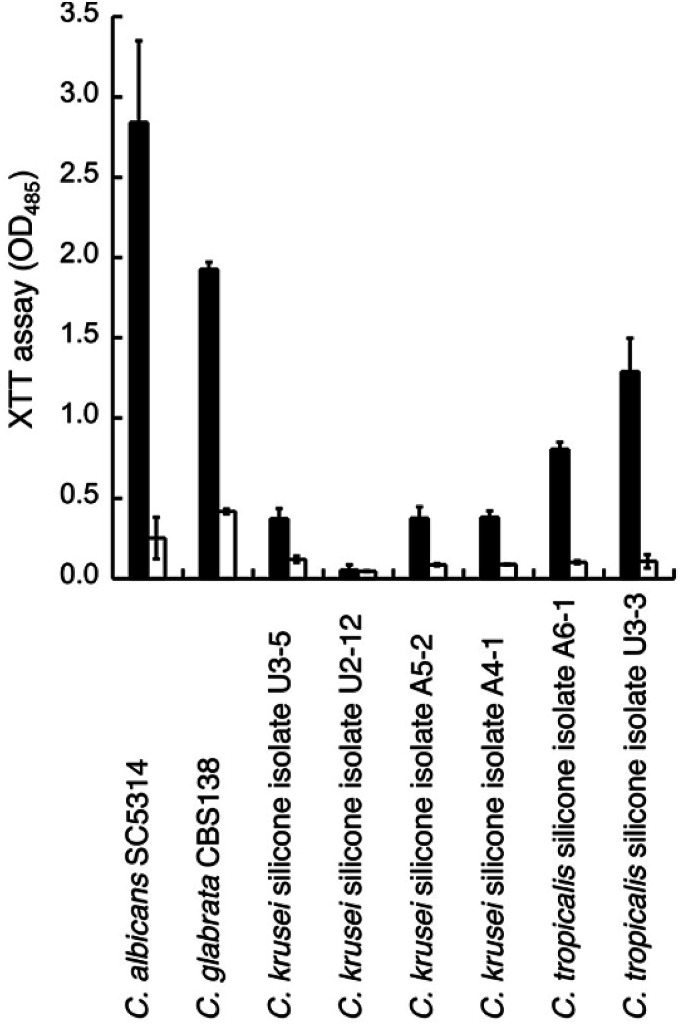
Treatment of mature (48 h) biofilm of several *Candida* species without (filled columns) or with 0.5 g mL^−1^ of GDL (unfilled columns). The biofilm was measured with XTT assay. Optical density was measured at 485 nm. All experiments were performed in triplicate.

We thus tested the effect of GDL on the viability of mature biofilms formed by *C. glabrata* sequenced strain (CBS138) and clinical isolates from biofilm of silicon voice prosthesis (2 strains of *C. tropicalis* and 4 strains of *C. krusei*) obtained from Atos Medical AB. Their biofilm levels were estimated using the XTT assay. The medium was removed from mature biofilm (grown for 48 h) and replaced with fresh medium with addition of GDL (0.5 g mL^−1^) followed by incubation for 24 h. Fresh medium without addition of GDL was used as control. For most strains, the biofilm formation was lowered upon addition of GDL (Fig. 7).

The effect shown by high concentrations of GDL is probably mediated through cell wall damage as the cells exposed to GDL had also lower viability on the medium with calcofluor white compared to that supplemented with osmotic stabilizer (0.5 M sucrose) and compared to the untreated cells on these media (ESI†).

## Discussion

β-1,3-Glucans, *i.e.* polysaccharides consisting of glucopyranose units coupled with β-1,3-glycosidic linkages, are major cell wall components of most pathogens. While the cell wall of *C. albicans* consists mainly of β-1,3-glucans, the corresponding biofilm consists mainly of a matrix of branched mannan-β-1,6-glucan conjugates (MGCx).^18^

β-1,3-Glucanases, *i.e.* enzymes that can hydrolyze β-1,3-glucans, are vital for cell wall remodeling in the build-up of biofilm. Furthermore, recent research has shown that β-1,3-glucanases are highly important for the stimulation of hyphal growth, *i.e.* the morphological transition between planktonic and hyphal forms and their effect was shown to be dose-dependent.^19^ The signalling pathway of glucanase-induced hyphal formation is mediated through cAMP and a major transcription factor of filamentation, Efg1, as the *efg1* mutant is unable to form hypha.

GDL is a known transition state-like inhibitor of β-glycosidases, such as β-1,3-glucanase. GDL forms a half chair in a complex with the enzyme active site and the electronic distribution of the carbonyl group mimic a positive charge at the anomeric carbon.^20^ Isolated β-1,3-glucanase from *C. albicans* showed an optimum pH of 5.5–5.6 and was inactivated above pH 6.5 or below 5.0. Furthermore, this enzyme was non-competitively inhibited by GDL, with a *K*_i_ value of 1.34 mM.^21,22^ We conclude that GDL exhibits dual action, *i.e.* both by lowering the pH, which inactivates the glucanase, as well as by non-competitive inhibition of the glucanase.

*C. albicans* often inhabits glucose limited niches, such as the vagina. However, the vagina is rich in other carbon sources, such as lactate, which can be used by the fungus. Although the lactate exposure can reduce the *C. albicans* hyphae formation, and cause formation of thinner β-1,3-glucan layers,^23^ it results in higher resistance of *C. albicans* against hyperosmotic stress or treatment with the antibiotic amphotericin B, compared to cells grown in glucose rich media. Recent findings showed that the lactate exposure also results in β-glucan masking, which makes *Candida* not recognizable by immune cells.^24^ This makes lactic acid less attractive for biofilm treatment.

It is reasonable to assume that GDL functions as an indicator of a favorable environment for several pathogens, such as *C. albicans*, which favors yeast-form cells over hyphae, with reduced biofilm formation as the result. Gluconic acid is produced by many bacteria as the product of glucose oxidation. In addition, gluconic acid, as well as the corresponding lactones, can be used by yeasts and other eukaryotes in the pentose phosphate pathway. Prokaryotes metabolize it by Entner–Doudoroff pathway. Interestingly, it has been shown that the addition of gluconic acid strongly diminished the biofilm formation of *Vibrio cholerae*,^25^ and the lactone was found to reduce the hyphae elongation in the fish mould pathogen *Saprolegnia monoica*.^26^

## Conclusions

**Fig. 8:**
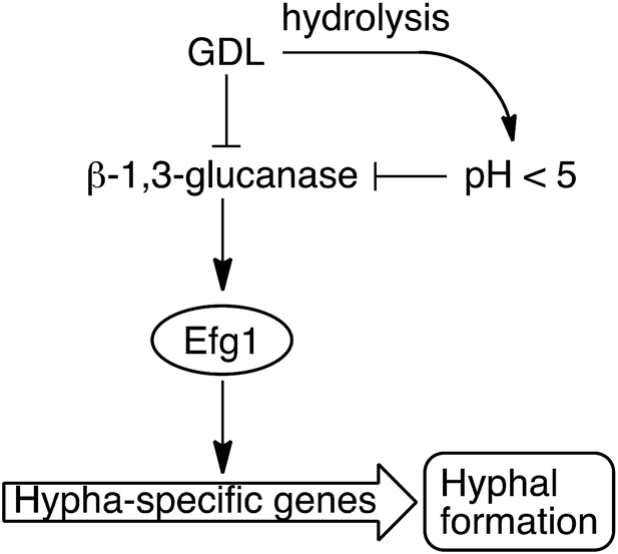
GDL and low pH, as a result of hydrolysis of GDL, inhibit the β-1,3-glucanase and consequently, less hyphae are formed.

We have shown that the biofilm formation in *C. albicans* and other *Candida* species can be significantly reduced by a combination of lowered pH and addition of GDL. We hypothesize that GDL, as well as functioning as a pH-lowering agent, inhibits β-1,3-glucanases. The lowered activity of β-1,3-glucanase results in lowered activity of Efg1, which in turn results in less activation of hypha-specific genes and consequently less hyphal formation (Fig. 8).^7^ Future investigation are needed to verify which gene expressions that contribute to the observed phenotype.

## Conflicts of interest

OS, HS, UE and SM have filed patent applications and hold patents in relation to the present work. OS, HS, UE and SM are shareholders of Gedea Biotech AB, a company with relationships to the present work.

## Supplementary Material

RA-009-C9RA01204D-s001

RA-009-C9RA01204D-s002
