# Supplementary material for: The use of polyhydroxylated carboxylic acids and lactones to diminish biofilm formation of the pathogenic yeast Candida albicans
Source: RSC Adv. 2019 Apr 9;9(19):10983–9. doi: 10.1039/c9ra01204d (PMC9062608; doi:10.1039/c9ra01204d)
Supplement: RA-009-C9RA01204D-s001 [file RA-009-C9RA01204D-s001.pdf]

Electronic Supplementary Material (ESI) for RSC Advances.
This journal is © The Royal Society of Chemistry 2019

Supporting Information

The use of polyhydroxylated carboxylic acids and lactones to diminish biofilm formation of the pathogenic yeast *Candida albicans*

Olena P. Ishchuk,[a] Olov Sterner,[b] Helena Strevens,[c] Ulf Ellervik,[b] Sophie Manner[b*]

[a] Department of Biology, Lund University, Sölvegatan 35, SE-223 62 Lund, Sweden.

[b] Centre for Analysis and Synthesis, Centre for Chemistry and Chemical Engineering, Lund University, P.O. Box 124, SE-221 00 Lund, Sweden.

[c] Department of Obtsetrics and Gynaecology, Skånes Universitssjukhus, SE-221 85 Lund, Sweden.

* Corresponding author. *E-mail address:* sophie.manner@chem.lu.se.

Sensitivity to calcofluor white as indicator of cell wall damage

To deduce cell wall damage, cells from biofilm experiments were plated onto YPD solid media with calcofluor white at 10 and 70 μg/mL with or without addition of 0.5 M sucrose (osmotic stabilizer) and incubated at 37 °C.

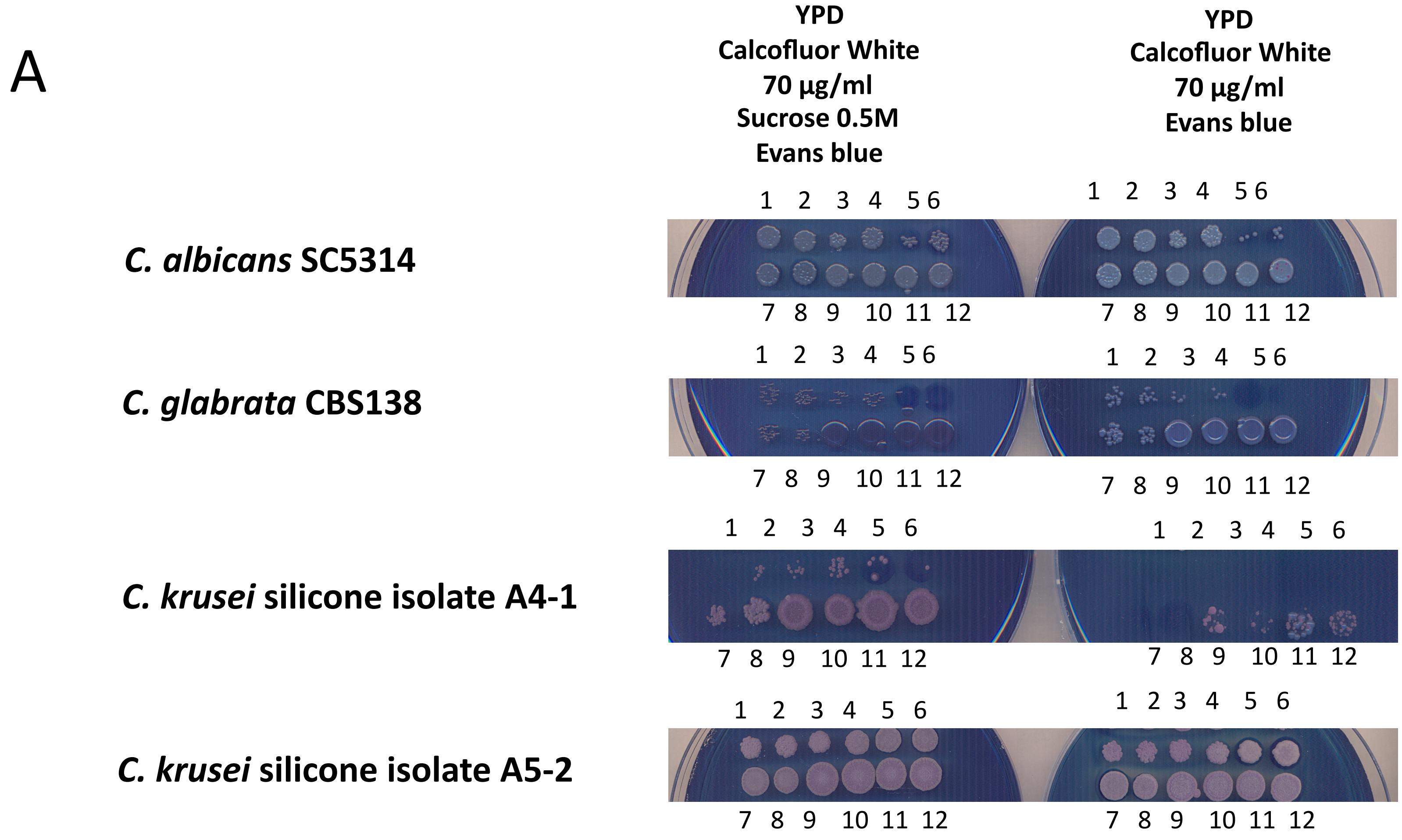
A
YPD
Calcofluor White
70 µg/ml
Sucrose 0.5M
Evans blue
YPD
Calcofluor White
70 µg/ml
Evans blue
C. albicans SC5314
C. glabrata CBS138
C. krusei silicone isolate A4-1
C. krusei silicone isolate A5-2
1 2 3 4 5 6
7 8 9 10 11 12

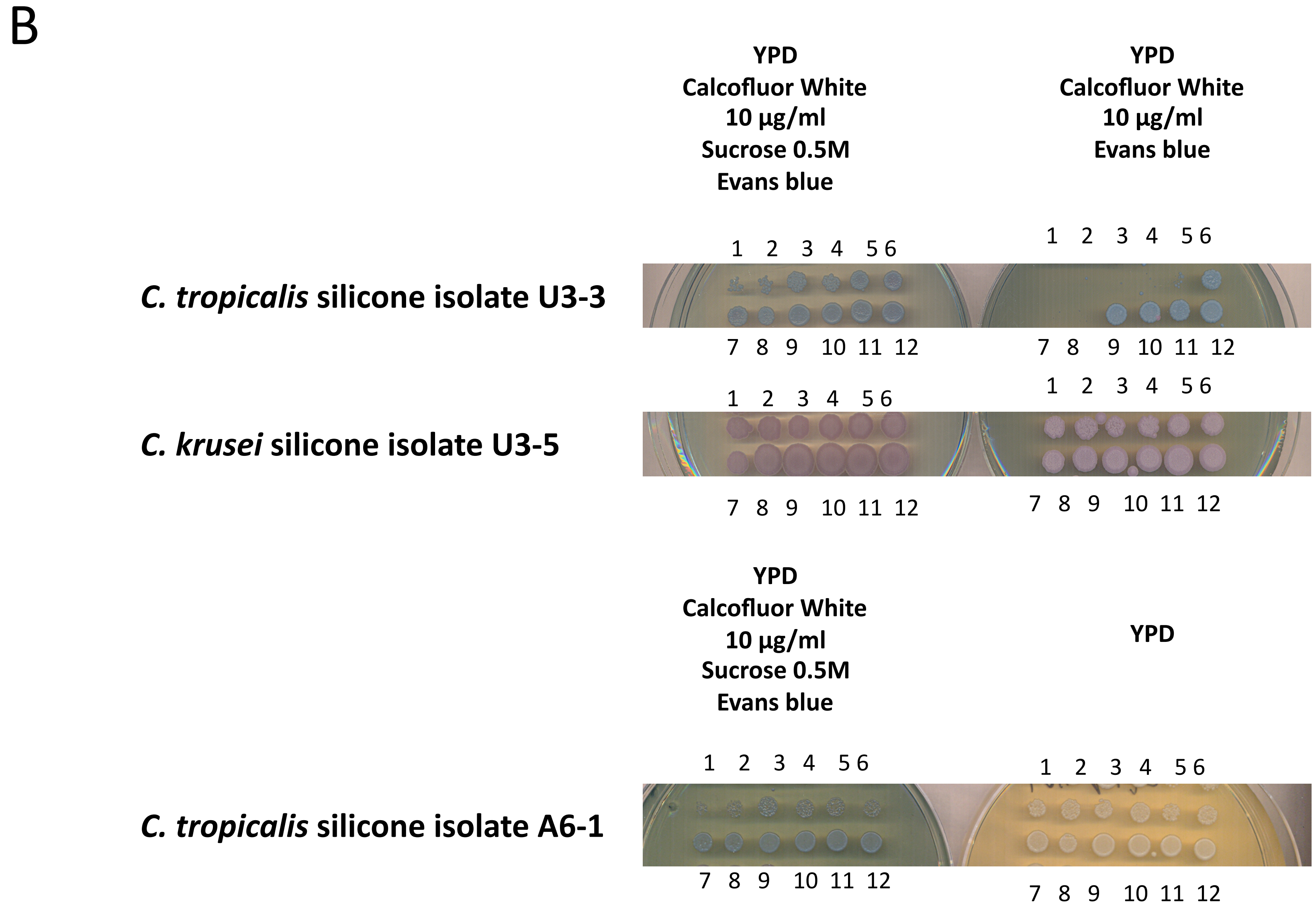
B
YPD
Calcofluor White
10 μg/ml
Sucrose 0.5M
Evans blue
YPD
Calcofluor White
10 μg/ml
Evans blue
C. tropicalis silicone isolate U3-3
C. krusei silicone isolate U3-5
YPD
Calcofluor White
10 μg/ml
Sucrose 0.5M
Evans blue
YPD
C. tropicalis silicone isolate A6-1
1 2 3 4 5 6
7 8 9 10 11 12
